# Gene expression profiling of alveolar soft-part sarcoma (ASPS)

**DOI:** 10.1186/1471-2407-9-22

**Published:** 2009-01-15

**Authors:** Luke H Stockwin, David T Vistica, Susan Kenney, David S Schrump, Donna O Butcher, Mark Raffeld, Robert H Shoemaker

**Affiliations:** 1 Drug Mechanisms Group, Developmental Therapeutics Program, SAIC-Frederick Inc., National Cancer Institute at Frederick, Frederick, MD 21702, USA; 2 Screening Technologies Branch, Developmental Therapeutics Program, National Cancer Institute at Frederick, Frederick, MD 21702, USA; 3 Laboratory of Pathology, National Cancer Institute, Bethesda, MD 20892, USA; 4 Thoracic Oncology Section, Surgery Branch, Center for Cancer Research, National Cancer Institute, Bethesda, MD 20892, USA; 5 Pathology/Histotechnology Laboratory, SAIC-Frederick Inc., National Cancer Institute at Frederick, Frederick, MD 21702, USA

## Abstract

**Background:**

Alveolar soft-part sarcoma (ASPS) is an extremely rare, highly vascular soft tissue sarcoma affecting predominantly adolescents and young adults. In an attempt to gain insight into the pathobiology of this enigmatic tumor, we performed the first genome-wide gene expression profiling study.

**Methods:**

For seven patients with confirmed primary or metastatic ASPS, RNA samples were isolated immediately following surgery, reverse transcribed to cDNA and each sample hybridized to duplicate high-density human U133 plus 2.0 microarrays. Array data was then analyzed relative to arrays hybridized to universal RNA to generate an unbiased transcriptome. Subsequent gene ontology analysis was used to identify transcripts with therapeutic or diagnostic potential. A subset of the most interesting genes was then validated using quantitative RT-PCR and immunohistochemistry.

**Results:**

Analysis of patient array data versus universal RNA identified elevated expression of transcripts related to angiogenesis (ANGPTL2, HIF-1 alpha, MDK, c-MET, VEGF, TIMP-2), cell proliferation (PRL, IGFBP1, NTSR2, PCSK1), metastasis (ADAM9, ECM1, POSTN) and steroid biosynthesis (CYP17A1 and STS). A number of muscle-restricted transcripts (ITGB1BP3/MIBP, MYF5, MYF6 and TRIM63) were also identified, strengthening the case for a muscle cell progenitor as the origin of disease. Transcript differentials were validated using real-time PCR and subsequent immunohistochemical analysis confirmed protein expression for several of the most interesting changes (MDK, c-MET, VEGF, POSTN, CYP17A1, ITGB1BP3/MIBP and TRIM63).

**Conclusion:**

Results from this first comprehensive study of ASPS gene expression identifies several targets involved in angiogenesis, metastasis and myogenic differentiation. These efforts represent the first step towards defining the cellular origin, pathogenesis and effective treatment strategies for this atypical malignancy.

## Background

Alveolar soft-part sarcoma (ASPS) is an extremely rare (1 diagnosis per 10 million population per year) soft tissue sarcoma with an indolent course and generally poor prognosis [1-3]. The disease manifests in young adults (15–35 years), where it is usually diagnosed from a painless mass in the lower limbs, head or neck [1,4]. ASPS is surprisingly slow growing with a clinical course averaging 15 years, where late stage disease is associated with metastasis to multiple sites including the lungs, bone, lymph nodes and brain [4]. The protracted ontogeny of ASPS makes it relatively resistant to classical chemotherapy and current treatment options are limited to surgical resection and localized radiotherapy.

Histologically, ASPS is composed of organoid nests of polygonal tumor cells encompassed by a dense capillary vasculature, giving rise to the 'alveolar' appearance [1,2]. In spite of several reports suggesting ASPS arises from a myogenic progenitor, the tissue ontogeny of ASPS is still contested, primarily because consistent detection of muscle markers (e.g. MYOD1 or MYOG) has not been demonstrated [5,6]. ASPS cells also contain numerous periodic acid Schiff-positive, diastase-resistant crystalline structures, recently identified as containing monocarboxylate transporter 1 (MCT1) and the cellular chaperone CD147 [7].

At the cytogenetic level, ASPS exhibits a conserved abnormality in the form of an unbalanced translocation der(17)t(X;17)(p11;q25) which fuses the N-terminal region of the alveolar soft part locus gene (*ASPL*), located at 17q25, to the C-terminal region of the transcription factor E3 (*TFE3*), located at Xp11. Two alternative fusion junctions have been observed resulting in the expression of two distinct fusion transcripts, *ASPL-TFE3 *type 1 and type 2 [8]. TFE3 is a ubiquitous basic helix-loop-helix (bHLH) leucine zipper transcription factor, which binds positive strand e-box motifs [CANNTG] [9]. High-affinity interactions with DNA occur when TFE3 is present as a homodimer or when combined with other bHLH factors including MITF, TFEB and TFEC [10]. Conversely, ASPL has only been partially characterized as a tethering protein that forces retention of GLUT4-containing vesicles in the cytoplasm in the absence of insulin [10,11].

The current consensus is that aberrant TFE3 transcriptional activity modulated by the ASPL fusion partner is likely responsible for elements of ASPS pathogenesis. Further support for the primacy of TFE3 comes from the detection of alternate TFE3 fusion proteins (PRCC-TFE3, PSF-TFE3, NONO-TFE3) in a subset of renal carcinomas [12-15]. As regards novel properties of ASPL-TFE3, one study using an anti-TFE3 antibody demonstrated 'aberrantly strong' nuclear staining in ASPS patients suggesting either enhanced nuclear trafficking, protein stability or expression levels [16]. More recently, fusion proteins containing TFE3 have been shown to directly activate transcription of *c-MET *(Hepatocyte growth factor receptor) through a consensus e-box [17]. Given that c-MET plays an important role in cell survival and angiogenesis, this important observation forges a link between ASPL-TFE3 and disease pathogenesis, intimating that genes with similar promoter elements may also be activated [18].

Therefore, as a slow growing yet highly vascular sarcoma, ASPS is an intriguing candidate for gene expression analysis. In this study, freshly isolated patient RNA was used to generate a transcriptomic profile for ASPS. Results demonstrated elevated expression for factors involved in angiogenesis including: angiopoietin-like 2 (*ANGPTL2*), the HGF receptor (*c-MET*), Hypoxia-Inducible factor 1α (*HIF1A*), midkine (*MDK*) and vascular endothelial growth factors A/B (*VEGF/VEGFB*). Further exploration identified muscle-specific beta 1 integrin binding protein (*ITGB1BP3/MIBP*), muscle-specific RING finger protein 1 (*TRIM63*) and Myogenic factors 5 and 6 (*MYF5/6*) as potential ASPS markers. Overall, these data identify molecular targets with the potential to aid the diagnosis and treatment of ASPS.

## Methods

A list of antibody sources, dilutions, retrieval conditions and positive control tissues used in immunohistochemical analysis is provided [see Additional file 1]. Unless otherwise indicated in the following methods, all other chemicals and inhibitors were from Sigma (St. Louis, MO).

### ASPS diagnosis and RNA isolation

Tumors were obtained from surgery following informed consent under NCI clinical research protocol 05-C-N138, approved by the U.S. National Cancer Institute (NCI) Institutional Review Board (IRB), and with the assistance of the Alliance Against Alveolar Soft-Part Sarcoma (TAAASPS). This research is in compliance with the Helsinki Declaration for conduct of research utilizing human subjects. A representative tumor sample (0.5–1.0 cm) was fixed in buffered 10% formalin for histopathology. The following criteria were employed for the diagnosis of ASPS: the presence of organoid nests of cells surrounded by endothelial-lined vascular channels in H&E stained material [1]; cytoplasmic PAS positive crystals following digestion with diastase [7]; the presence of the ASPL-TFE3 type 1 or type 2 fusion transcript [8], nuclear staining with antibodies to the transcription factor TFE3 [16] and to either the ASPL-TFE3 type 1 or type 2 fusion protein [19]. For isolation of RNA, a portion of tumor tissue was immediately placed in RNA later solution (Ambion, Austin, TX) and stored at 4°C. Total RNA was prepared using the RNeasy Fibrous Midi Kit (Qiagen, Valencia, CA) according to the manufacturer's instructions. The concentration of RNA was determined on a Nanodrop spectrophotometer, and the A260/A280 values were greater than 1.9. The integrity and size distribution of RNA were evaluated on 1% denaturing NuSieve agarose gels (FMC, Rockland, ME) followed by ethidium bromide staining.

### Reverse transcription

Total RNA was isolated from cells using the RNeasy mini kit (Qiagen, Valencia, CA) and reverse transcribed using Omniscript RT according to the manufacturers' instructions. A standard reaction comprised 2 μg total RNA, 0.5 mM each dNTP, 2 μM random decamers (Ambion, Austin, TX) and 4 units of reverse transcriptase in 20 μl total volume of 1× RT buffer. The reaction was allowed to proceed for 90 min at 37°C and the cDNA product diluted to 1 μg/ml and stored at -80°C.

### Microarray data acquisition

Microarray data was collected at Expression Analysis, Inc. (Durham, NC) using the GeneChip^® ^Human U133 plus 2.0 Array (Affymetrix), according to standardized operating procedures. Overall, analysis was performed on RNA from seven ASPS patients and universal RNA, where all samples were analyzed in duplicate. The universal reference RNA (Clontech) is a mixture of RNAs from a collection of adult human tissues (male and female) representing a broad range of expressed genes. Affymetrix array data files (.CEL) have been deposited with the Gene Expression omnibus http://www.ncbi.nlm.nih.gov/geo/query/acc.cgi?acc=GSE13433.

### Microarray data analysis

The Genesifter suite (VizX labs, Seattle, WA) was used for analysis of microarray data. In brief, compressed .CEL files containing array data were loaded into the Genesifter web portal http://www.genesifter.net. Using the advanced upload function, probe-level data was then compiled, normalized and transformed using GC-RMA [20]. Pairwise analysis was then performed comparing universal RNA arrays with ASPS patient arrays. The following criteria were applied to filter the differentially expressed transcript list, a fold change of >3 and a Wilcoxon rank sum test where p < 0.05 with Benjamini-Hochberg estimation of false discovery rate (FDR). The list of differentially regulated transcripts was then exported into excel for gene ontogeny analysis.

### Quantitative RT-PCR

SYBR Green chemistry was used to detect primer specific amplicons. For each reaction, 12.5 μl Quantitect SYBR Green PCR mastermix (Qiagen, Valencia, CA) was diluted 1:2 in DNase free water containing 5 ng cDNA and 1 μM of specific primer pair. Reactions were performed in triplicate and universal 18S RNA primers (Ambion, Austin, TX) were used to normalize cDNA amplification. The fluorochrome ROX, included in the PCR mastermix, was used as a passive reference. Reactions were performed using an ABI7500 thermocycler (Applied Biosystems, Foster City, CA). Cycling conditions consisted of a single 10 min/95°C activation step followed by 50 cycles of 95°C/15 s, 60°C/60 s and 72°C/60 s with fluorescence measurements taken in the elongation step of each cycle. Fold changes and p values were then calculated by copying CT data into a Superarray analysis template (Superarray Inc. Frederick, MD). For each primer pair melting curve analysis was used to confirm the presence of a single amplicon. Sequences of primers used in quantitative RT-PCR are provided [see Additional file 2].

### Immunohistochemistry

In brief, tissue sections (5 μm) affixed to glass slides, were deparaffinized and treated for 15 minutes in methanol containing 0.6% H_2_O_2 _followed by either 1 mM EDTA (pH 8.0) or 10 mM citrate buffer (pH 6.0) in a microwave vacuum histoprocessor (RHS-1; Milestone, Italy). The temperature was gradually increased to 100°C over 10 minutes and maintained at 100°C for an additional 10 minutes. Sections were cooled, rinsed sequentially in H_2_O and PBS for 10 minutes each. Primary antibodies, used at dilutions indicated [see Additional file 1] were overlayed onto the tissue sections and incubated either overnight or for 30 min at room temperature. Sections were rinsed in PBS for 10 minutes and incubated with the appropriate secondary antibody for 30 minutes. Sections were rinsed in PBS for 10 minutes and processed according to instructions detailed in the detection kits.

## Results

### Microarray analysis

For seven patients with confirmed ASPS, fresh tumor specimens were obtained directly after surgery, total RNA was extracted, quality assured and then reverse transcribed to cDNA. Variables for these tumors in terms of sex, age, primary/metastatic disease and ASPL-TFE3 fusion type (1/2) are shown in Table 1. After reverse transcription, the seven cDNAs were each hybridized to duplicate high-density U133 plus 2 microarrays (~47,400 transcript). Two additional arrays, hybridized to universal cDNA (Promega) were included as standards. The choice of universal RNA normalization, as opposed to normalizing against a single normal tissue or alternate pooled sarcoma ensured that an relatively unbiased (basal) gene expression profile was generated. Analysis of combined ASPS patient arrays versus universal RNA was performed using the pairwise analysis function within GeneSifter (VizX labs), where the duplicate universal RNA arrays (control group) were analyzed against duplicate arrays for all seven ASPS patients (test group). A list of differentially expressed transcripts was then generated according to the following criteria; GC-RMA normalization, <0.05 adjusted p-value, Benjamini and Hochberg false discovery estimates with > +/- 3 fold changes in expression. The generated output consisted of 7367 differentially regulated transcripts, where 4731 (64.2%) were downregulated and 2636 (35.7%) showed upregulated expression [see Additional file 3].

**Table 1:** ASPS Patient Demographics

Patient	Age	Sex	ASPL-TFE3 Fusion Type	Primary or Metastasis
1	26	M	2	P (Thigh)
2	39	F	1	M (Mandible)
3	25	M	2	P (Hip)
4	15	F	2	M (Lung)
5	14	M	2	P (Calf)
6	27	F	1	P (Thigh)
7	22	M	2	M (Lung)

ASPL-TFE3 fusion type determined using RT-PCR according to Ladanyi *et al*. 2001 [8]

### Validation and gene ontology analysis

**Figure 1:**
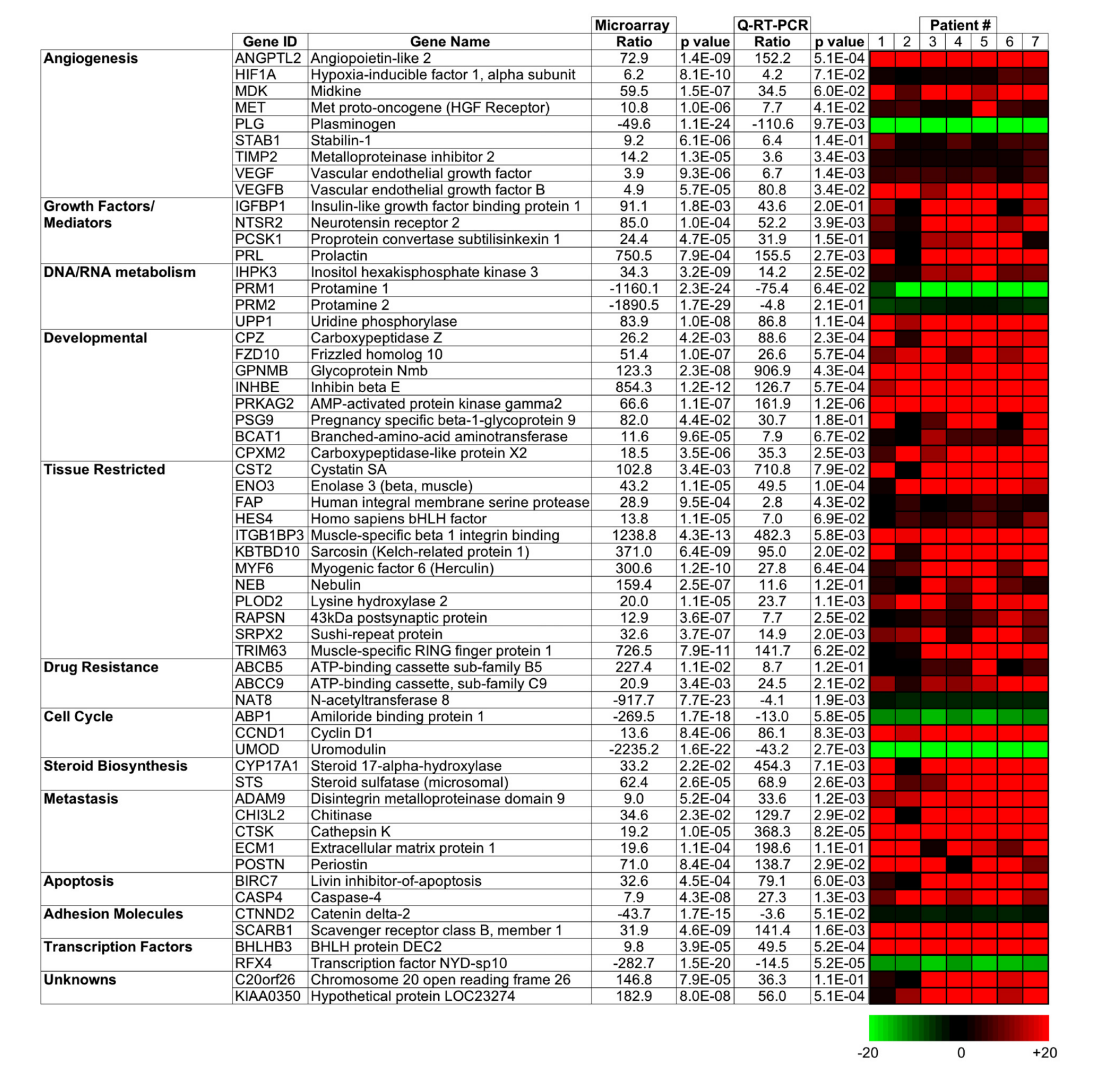
**Validation of Microarray Data for selected transcripts by Quantitative RT-PCR**. For a subset of genes identified as potentially interesting from GO analysis, quantitative RT-PCR analysis was performed to validate the extent and direction of change. Results are shown subdivided into molecular function, with fold change values for both microarray and quantitative RT-PCR shown along with heat map representation of individual patient values with +/- 20 fold limits.

Microarrary data was then interrogated with reference to gene ontology (GO) databases with the overall aim of identifying a subset of genes for validation with quantitative RT-PCR. Transcripts with potential disease relevance or drugability, such as those implicated in angiogenesis, metastasis, proliferation and drug resistance were of particular interest. Reference databases used to mine gene ontology information included Ingenuity Pathways Analysis http://www.Ingenuity.com, Entrez Gene http://www.ncbi.nlm.nih.gov, Genecards http://www.genecards.org/index.shtml and Symatlas http://symatlas.gnf.org/SymAtlas/. A subset of relevant genes was then validated using SYBR-green based quantitative RT-PCR. In total, primers were designed for 61 genes and expression relative to universal RNA determined for all seven ASPS patient samples. A summary of results from both microarray analysis and quantitative RT-PCR, subdivided into molecular function is shown in Figure 1. Results demonstrated concordance in direction of change between techniques with acceptable p-values, supporting differential regulation of the gene subset. However, for several transcripts a significant discrepancy was evident in the extent of change between the different techniques. Although some differences can be anticipated based on primer/probe specificity, methods for calculating fold change and dynamic range of detection, results for several transcripts were considerable (PRM1/2 and UMOD). For example, protamine 1 was downregulated 1160.1 fold by microarray versus 75.4 fold for RT-PCR. This result should be regarded as a 'zero' result for the microarray rather than a true representation of fold change and highlights a potential drawback in the use of universal RNA as a standard given that it favors extreme differentials that may reside outside of the quantitation range of a technique.

### Immunohistochemistry

**Figure 2:**
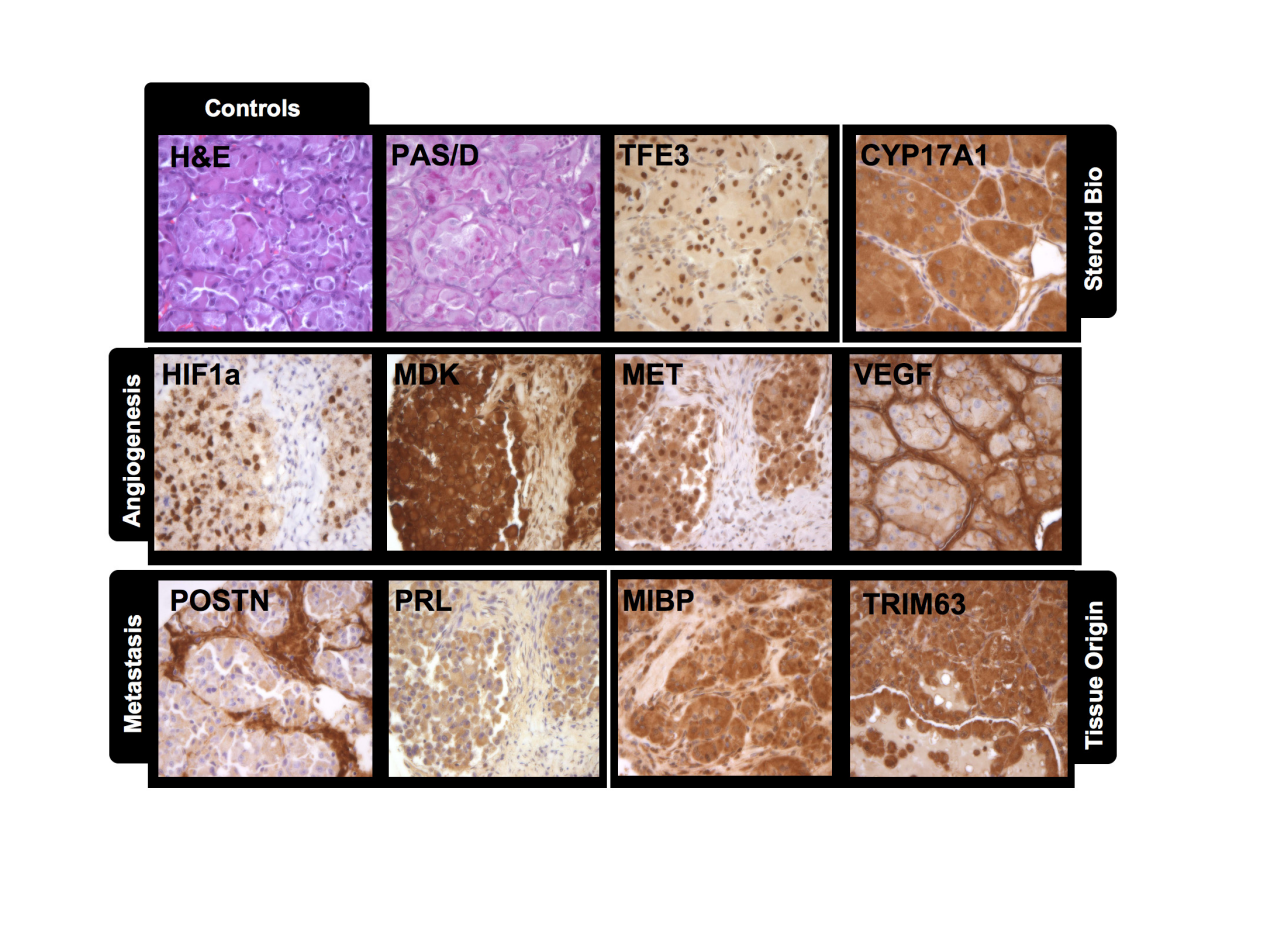
**Analysis of marker protein expression using an ASPS tissue array. Paraffin-embedded sections from seven ASPS tumors were arrayed on glass slides and analyzed by immunohistochemistry**. For all sections, ASPS origin was confirmed by detection of intracellular granules using periodic-acid Schiff diastase (PAS/D) staining and nuclear reactivity when stained with an anti-TFE3 antibody. Images shown are representative of staining patterns for the majority tumors. All Images were captured at 350 × magnification.

A tissue array of paraffin-embedded ASPS tumors was then constructed for immunohistochemical (IHC) analysis. Each array contained multiple sections from each of the seven patients, where ASPS origin was confirmed by staining with H&E, PAS/diastase and α-TFE3 antibody. Staining protocols were then established using several commercially available antibodies against targets identified during gene expression analysis. Representative examples of IHC staining patterns for each experimental antibody are shown in Figure 2. Results demonstrated that an antibody against HIF-1α showed nuclear localized staining consistent with the regulation of HIF-1α expression by hypoxia gradients. An Anti-VEGF antibody produced intense and consistent staining of endothelial cells, highlighting the potential importance of this mediator in establishing ASPS morphology and potentially also in disease progression. A similar staining pattern was evident using antibodies against the secreted protein Periostin (POSTN), suggesting that endothelial cells are either the source of or ultimate target of this molecule. Conversely, midkine (MDK) staining was found across all patients and localized to the cytoplasm of the ASPS tumor cells themselves. Interestingly, for c-MET, all ASPS tumors were positive with expression localized to both cytoplasm and nucleus, which is consistent with it's recognized cycling between these two cellular compartments. Some of the most intense staining was observed for an antibody against CYP17A1, which localized to tumor cell cytoplasm. Lastly, antibodies against the muscle-specific proteins ITGB1BP3/MIBP and TRIM63 reacted consistently with ASPS tumor sections with cytosolic localization. These experiments suggest that detection of mRNA reflects, at least qualitatively, expression of protein. More importantly, immunohistochemistry data provides confidence that biological inferences can be drawn from the broader transcriptomic analysis.

## Discussion

**Figure 3:**
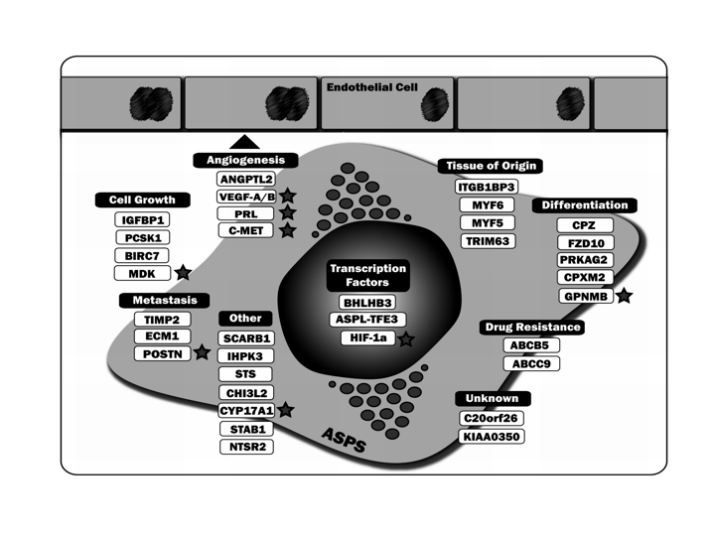
**Potential ASPS diagnostic and therapeutic targets identified during the study**. Established therapeutic targets or those currently undergoing development are marked with a star.

Our current understanding of ASPS disease origin, pathogenesis and effective treatment strategies is rudimentary. In this study, we began to address these issues by generating a genome-wide ASPS transcriptome using freshly isolated patient RNA. The quality of target RNA is a central issue in successful gene expression analysis, especially when using high-feature microarrays. Therefore, the decision was taken to focus efforts on RNA freshly isolated from a small number of ASPS patients rather than utilizing degraded RNA from archived paraffin-embedded specimens. Results demonstrated that ASPS has a molecular signature encompassing genes with therapeutic and diagnostic potential (Figure 3). Similarly, identification of elevated expression for several transcripts provided clues towards the cellular origin of disease.

Altered expression of transcripts involved in the response to hypoxia and angiogenesis suggests that these pathways are active in ASPS and provides some of the most interesting candidates for therapeutic intervention. The greatest increase in angiogenesis-related genes was seen for angiopoietin-like 2 (ANGPTL2). Angiopoietins are hypoxia-regulated members of the vascular endothelial growth factor (VEGF) family, where ANGPTL2 acts as an endothelial survival factor that does not bind TIE1 or TIE2 [21]. Increased expression was also observed for the master transcriptional regulator of the response to hypoxia, HIF-1α (HIF1A), which is interesting given that regulation of HIF-1α occurs predominantly through a post-translational mechanism [22]. Similarly, enhanced expression of Midkine (MDK), a hypoxia-induced growth factor involved in cell survival and migration, provides a potential target for inhibition [23]. Here, we also confirm consistent increases in expression of the hepatocyte growth factor receptor (MET). In a recent study, ASPL-TFE3 and other TFE3 fusion proteins were shown to bind and strongly activate the MET promoter [17]. Furthermore, in cells expressing TFE3, inhibition of MET using siRNA or a small molecule MET inhibitor impaired tumor growth. A similarly important observation concerned elevated expression of two members of the VEGF family, VEGF-A and VEGF-B, supporting the utility of inhibitors such as Bevacizumab in the treatment of ASPS [24]. Increased expression of Stabilin (STAB1) may also contribute towards an angiogenic phenotype, given that this scavenger receptor is expressed on endothelial cells and inhibition with monoclonal antibodies has been shown to prevent cord formation *in vitro *[25]. In a recent study, RNA isolated from formalin-fixed paraffin-embedded ASPS tumor material was subjected to focused microarray analysis with emphasis on genes involved in angiogenesis [26]. Overall, 18 genes were found to be upregulated >1.5 fold with 8 regulated >3 fold. Cross-comparison showed that 7/18 of these genes (MDK, PTN, JAG1, TGFB1, NRP1, HIF1A and TIMP2) were represented in our transcriptome (which utilizes a 3-fold cut-off). These results are noteworthy as they confirm several of our own observations and highlight a possible role for JAG1. Overall, identification of several angiogenic mediators (Angiopoietin-like 2, c-MET, HIF-1α and VEGF) provides a molecular framework for ASPS vascular histology and supports the use of anti-angiogenic agents in treatment [24].

Increased expression of several mitogenic factors was also noted. One example was Prolactin (PRL), a 23 kDa peptide hormone implicated in a diverse range of processes including lactation and cell proliferation [27]. Prolactin is recognized as a breast cancer cell survival factor and prolactin inhibitors demonstrate the ability to inhibit cell proliferation *in vitro *and to slow tumor growth in mice [28,29]. Further transcripts involved in growth regulation showing upregulation included; insulin like growth factor binding protein 1 (IGFBP1), Neurotensin receptor 2 (NTSR2) and proprotein convertase subtilisin/kexin type 1 (PCSK1).

Several genes broadly implicated in metastasis showed elevated expression. Periostin (POSTN) is a 90 kDa disulphide-linked secreted protein which has homology to the axon guidance protein fasciclin I [30]. Periostin is expressed predominantly in fetal tissues and also in ovarian cancer, where it binds αVβ3/αVβ5 integrins and promotes cellular motility [31]. Neutralizing therapeutic antibodies directed against Periostin have already undergone pre-clinical evaluation, with promising results [32]. Extracellular matrix protein 1 (ECM1) meanwhile is a recently identified secreted glycoprotein, over-expressed in several epithelial malignancies, recognized as capable of promoting metastasis and angiogenesis [33]. A disintegrin and metalloproteinase domain 9 (ADAM9) is a membrane bound metalloprotease, which like the other ADAM family members plays a role in cellular invasion, metastasis and angiogenesis [34]. Along with proteolytic activity, ADAM9 appears to function as an adhesion molecule mediating interactions with αvβ5 integrin [35]. This protein is expressed in a variety of normal tissues and shows elevated expression in certain cancers and under conditions of cellular stress [36].

Aberrant expression was also noted for transcripts implicated in embryonic development. Pregnancy-specific glycoprotein 9 (PSG9) is a secreted glycoprotein belonging to the CEA family. In colorectal cancer, PSG9 also shows elevated expression [37]. However, the function of the antigen is largely unknown, although an immunological role has been suggested [38]. Equally, glycoprotein NMB (GPNMB) is a type I membrane protein with homology to the melanoma antigen pMEL17 [39]. This antigen has been identified at the plasma membrane of several malignancies including melanoma and glioma [39,40]. A humanized toxin-conjugated NMB antibody was shown to be effective in killing antigen-positive melanoma, suggesting that this therapeutic may have potential in the treatment of other NMB positive malignancies [41]. Enhanced expression was observed for Inhibin beta E chain (INHBE), a subunit which contributes towards the activin/inhibin pleotropic hormone axis. Inhibins are heterodimers of one α and one β subunit, whereas activins are homo- or heterodimers of β subunits only. As members of the extended TGF-β super family, these activins play a number of roles including cell proliferation, differentiation and apoptosis [42]. Two genes involved in steroid biosynthesis, cytochrome P450-17A1 (CYP17A1) and Steroid sulfatase (STS), also show increased expression. CYP17A1 is an enzyme that converts pregnenolone and progesterone to their 17- alpha-hydroxylated products whereas STS catalyzes the conversion of sulfated steroid precursors to estrogens.

With respect to defining ASPS cellular ontogeny, Symatlas identified a preponderance of muscle-restricted genes in the ASPS transcriptome (results not shown). Classical markers of skeletal muscle development (myogenin, myoblast determination protein 1, desmin and myoglobin) were not detected at the mRNA level, mirroring reports obtained elsewhere [6]. However, myogenic factors 5 and 6 (MYF5/6), important members of the myogenic basic helix-loop-helix (bHLH) family of transcription factors, were upregulated. Interestingly, myogenic factors such as these bind to the same E-box consensus motif [CANNTG] as TFE3 [43]. In terms of myogenic differentiation, evidence suggests that MYF5 is involved in progenitor cell proliferation whereas myoblast determination protein 1 and myogenin are concerned more with cell specialization and terminal differentiation [44]. A further compelling candidate for an ASPS marker was Muscle-Specific B1 Integrin Binding Protein (ITGB1BP3/MIBP). Expression of this transcript was significantly increased in ASPS relative to universal RNA (1238 up microarray; 482 up QPCR) and according to Symatlas is expressed primarily in skeletal muscle and heart. A possible link to ASPS pathogenesis may be inferred from one recent study showing that in myoblasts, ITGB1BP3 suppresses fusion and terminal differentiation [45]. This protein appears to function by selectively binding the α7β1 v laminin receptor and mediating reduced laminin cell adhesion and matrix deposition [46]. Similarly, muscle specific enolase 3 (ENO3) was upregulated in all patients. This enzyme is a HIF-1α inducible gene thought to be involved in striated muscle development and regeneration [47,48]. Increased expression was similarly observed for Muscle-specific RING finger protein 1 (TRIM63), an E3 ubiquitin ligase involved in the degradation of sarcomere-associated proteins such as troponin [49]. In combination with atrogin-1, this protein is thought to be involved in skeletal muscle atrophy [50]. A further upregulated skeletal muscle restricted transcript was Nebulin (NEB), a giant (800 kDa) protein that maintains the structural integrity of sarcomeres in skeletal muscle [51]. ASPS cells are reported to contain cytoplasmic granules containing monocarboxylate transporter 1 (MCT1) and the accessory protein Basigin (BSG/CD147) [7]. A search of our array data identified increased expression of MCT1 but not CD147.

A group of smooth muscle restricted transcripts were similarly upregulated. Fibroblast activation protein α (FAP) is a gelatinase implicated in tissue remodeling and tumor invasion expressed primarily in smooth muscle and also found in some melanoma cell lines [52]. Sushi-repeat-containing protein, X-linked 2 (SRPX2) is a poorly characterized protein expressed primarily in smooth muscle and neuronal tissue [53]. Finally, lysyl hydroxylase 2 (PLOD2) is responsible for the production of stabilizing hydroxylysine residues in collagens and shows highest expression in smooth muscle [54].

Therefore, detection of two myogenic regulatory factors MYF5/MYF6 along with several other muscle-specific transcripts including ITGB1BP3/MIBP and TRIM63 seems to favor a myogenic progenitor [44]. Several reports have explored the possible myogenic origin of ASPS [55-61]. Within these, there are conflicting reports of expression of the muscle-specific factor MyoD1 [58-60]. Our data suggest that MyoD1 is absent whereas family members MYF5 and MYF6 are expressed. We also concur with results showing that ASPS is myogenin negative [61]. Therapeutic targeting of muscle-specific protein may also have potential. For example, experiments using siRNAs are currently underway to determine whether the reported ability of ITGB1BP3/MIBP to inhibit myoblasts terminal differentiation also extends to ASPS cells [45].

## Conclusion

In conclusion, the work presented here comprises the first genome-wide microarray analysis of ASPS; generated using RNA isolated from tumors at the time of surgery. The approach used strongly suggests that for rare tumors such as ASPS, microarray analysis of small number of fresh tumor RNA samples is preferable to analysis of RNA from large numbers of archival specimens. Results identify targets with diagnostic/therapeutic potential and suggest a myogenic progenitor as the origin of disease. Our ongoing efforts towards classification of ASPS in the context of other soft-tissue sarcomas and the development of an ASPS cell line will further address the issue of cellular origin. An equally important question concerns the exact role of the ASPL-TFE3 fusion protein in tumorigenesis. The ability of fusion proteins containing TFE3 to transactivate e-box containing genes such as c-MET suggests that certain facets of the transcriptome are related to ASPL-TFE3 activity [17]. It is hoped that by making this array data publically available we will promote additional investigations centered on this unique malignancy and provide a useful reference dataset for the wider classification of soft-tissue sarcomas.

## Competing interests

The authors declare that they have no competing interests.

## Authors' contributions

LHS was responsible for microarray analysis, figure production and the final manuscript. DTV, DSS and RS were responsible for initiating the study along design, execution and manuscript preparation. SK was responsible for ASPS tumor sample acquisition, RNA preparation and cDNA synthesis. MR and DOB were responsible for immunohistochemistry. All authors have read and approved the final version of the manuscript.

## Pre-publication history

The pre-publication history for this paper can be accessed here:

http://www.biomedcentral.com/1471-2407/9/22/prepub

## Supplementary Material

Additional File 1 I**mmunohistochemistry: Antibody Source, Antigen Retrieval and Positive Controls.** Table of conditions used for Immunohistochemistry of ASPS tumor sections. Click here for file

Additional File 2 Primer sequences. Table of primer sequences used during SYBR green quantitative RT-PCR validation of ASPS patient cDNA. Click here for file

Additional File 3 **Differential Expression of ASPS Genes versus Universal RNA.** Genesifter output from pairwise analysis of all ASPS patient arrays versus universal RNA. Click here for file
