# Supplementary material for: Gene expression profiling of alveolar soft-part sarcoma (ASPS)
Source: BMC Cancer. 2009 Jan 15;9:22. doi: 10.1186/1471-2407-9-22 (PMC2635365; doi:10.1186/1471-2407-9-22)
Supplement: Additional File 1 — Immunohistochemistry: Antibody Source, Antigen Retrieval and Positive Controls. Table of conditions used for Immunohistochemistry of ASPS tumor sections. [file 1471-2407-9-22-S1.doc]

| **1AB** | **DILUTION** | **ANTIGEN RETRIEVAL** | **PROTEOLYSIS** | **TECHNIQUE/ KIT** | **FIXATIVE** | **CHROMAGEN** | **POSITIVE CONTROL** | **1AB MANUF.** | **1AB CAT. #** |
| --- | --- | --- | --- | --- | --- | --- | --- | --- | --- |
| CYP17A1 | 1:200 | HIER Citrate 20' | Not used | NRS/ 2Ab Rabbit a/Goat/ SA-HRP | 10% NBF | DAB | Human Ovary | Novus Biologicals | NB100-2842 |
| Enolase 3 | 1:50 | Not used | Not used | NHS/ 2Ab Horse a/Mouse/ SA-HRP | 10% NBF | DAB | Human Skeletal Muscle | Novus Biologicals | H00002027-A01 |
| HIF1-a | 1;2000 | MW EDTA pH 8.0 | Not used | Dako CSA II | 10% NBF | DAB | Human Renal Carcinoma | Novus Biologicals | NB-100-123 |
| Met | 1:1000 ON 4c | MW Citrate buffer | Not used | Vectastain Rabbit E | 10% NBF | DAB | Human Breast | Santa Cruz | sc-161 |
| MIBP | 1:50 | HIER EDTA 10' | Not used | NHS/ 2Ab Horse a/Mouse/ SA-HRP | 10% NBF | DAB | Human Skeletal Muscle | M&B Laboratories | K0099-3 |
| Midkine | 1:20 ON 4c | MW Citrate buffer | Not used | Vectastain Goat E | 10% NBF | DAB | Human Breast | R&D Systems | AF-258-PB |
| Periostin | 1:1000 | Not used | Not used | NGS/ 2Ab Goat a/Rabbit/ SA-HRP | 10% NBF | DAB | Human Breast Cancer | Abcam | ab14041 |
| Prolactin | 1:250 | Not used | Not used | Vectastain Rabbit E | 10% NBF | DAB | Human Pituitary | DAKO | A0569 |
| TFE3 | 1:10,000 ON 4C | MW EDTA pH 8.0 | Not used | 10% NRS/ Vectastain Goat E | 10% NBF | DAB | ASPS | Santa Cruz | sc-5958 |
| TRIM63 | 1:50 | Not used | Not used | NHS/ 2Ab Horse a/Mouse/ SA-HRP | 10% NBF | DAB | Human Skeletal Muscle | Novus Biologicals | H00084676-A01 |
| VEGF | 1:100 | MW EDTA pH 8.0 | Not used | 10% NRS/ Vectastain Goat E | 10% NBF | DAB | Human Pancreas | R&D Systems | AF-293-NA |
